# Diversification and Specialization of Plant RBR Ubiquitin Ligases

**DOI:** 10.1371/journal.pone.0011579

**Published:** 2010-07-14

**Authors:** Ignacio Marín

**Affiliations:** Instituto de Biomedicina de Valencia, Consejo Superior de Investigaciones Científicas, Valencia, Spain; Michigan State University, United States of America

## Abstract

**Background:**

RBR ubiquitin ligases are components of the ubiquitin-proteasome system present in all eukaryotes. They are characterized by having the RBR (RING – IBR – RING) supradomain. In this study, the patterns of emergence of RBR genes in plants are described.

**Methodology/Principal Findings:**

Phylogenetic and structural data confirm that just four RBR subfamilies (Ariadne, ARA54, Plant I/Helicase and Plant II) exist in viridiplantae. All of them originated before the split that separated green algae from the rest of plants. Multiple genes of two of these subfamilies (Ariadne and Plant II) appeared in early plant evolution. It is deduced that the common ancestor of all plants contained at least five RBR genes and the available data suggest that this number has been increasing slowly along streptophyta evolution, although losses, especially of Helicase RBR genes, have also occurred in several lineages. Some higher plants (e. g. *Arabidopsis thaliana*, *Oryza sativa*) contain a very large number of RBR genes and many of them were recently generated by tandem duplications. Microarray data indicate that most of these new genes have low-level and sometimes specific expression patterns. On the contrary, and as occurs in animals, a small set of older genes are broadly expressed at higher levels.

**Conclusions/Significance:**

The available data suggests that the dynamics of appearance and conservation of RBR genes is quite different in plants from what has been described in animals. In animals, an abrupt emergence of many structurally diverse RBR subfamilies in early animal history, followed by losses of multiple genes in particular lineages, occurred. These patterns are not observed in plants. It is also shown that while both plants and animals contain a small, similar set of essential RBR genes, the rest evolves differently. The functional implications of these results are discussed.

## Introduction

In eukaryotes, protein ubiquitination is a key biochemical mechanism that is involved in multiple cellular processes, from the control of protein levels to the regulation of gene expression [1]–[4]. Given the wide functional implications of the system, there is a great interest in understanding in detail the groups of proteins which are involved in the process and in the regulation of ubiquitination. Of particular significance are ubiquitin ligases (E3s), the proteins that attach ubiquitin to the substrates, given that they are very numerous, structurally diverse and, most significantly, they provide specificity to the ubiquitination process [4]. A particular family of ubiquitin ligases, called RBR, has recently received a significant degree of attention, particularly due to the involvement of mutations in the gene that encodes one of them, parkin, in the genesis of Parkinson's disease (reviewed in [5], [6]). The RBR ubiquitin ligases are characterized by containing a supradomain, known as RBR signature, which consists in three consecutive protein domains. The most N-terminal, often called RING1, is a typical RING finger. RING fingers are present in many ubiquitin ligases and have an essential role in facilitating the transfer of ubiquitin to the substrate. However, it is only in RBR proteins that the RING finger is followed by two additional, characteristic domains. The first, named IBR (“In-between-rings”), consists in two consecutive zinc-binding domains [7]. The second, C-terminal, RING2 domain, is somewhat similar in sequence but structurally different from a canonical RING finger [8], [9]. The RBR signature is rich in conserved cysteines and histidines, with a pattern that can be summarized as C_3_HC_4_ - C_6_HC - C_3_HC_4_, respectively corresponding to the RING1, IBR and RING2 domains. This RBR signature is so characteristic that it is very simple to establish whether a particular protein is an RBR ubiquitin ligase. This has allowed for precise analyses of the origin and evolution of the RBR family (see the works of my group: [5], [8], [10], [11]). In those studies, it was established that RBR proteins appeared very early in eukaryotic evolution. They have been detected so far in all eukaryote groups for which sequence data are available. Moreover, detailed phylogenetic analyses allowed establishing a classification of RBR proteins into 14 main subfamilies [10], [11]. All the proteins of a particular subfamily are characterized by having very similar sequences and, often, by containing additional, subfamily-specific, protein domains. Just three of these subfamilies, called Ariadne, ARA54 and Helicase (also called “Plant I”, given that it was first found in plants), have been detected in both unikonts and bikonts, implying a very ancient origin. The rest are restricted to particular lineages. For example, the Parkinson-disease related gene *parkin*, mentioned above, belongs to an animal-specific subfamily, which has been called also parkin.

In a recent study, I analyzed in great detail the evolution of RBR ubiquitin ligases in animals [11]. Animals contain proteins of many RBR subfamilies which are not found in other organisms. It turned out that most of those subfamilies emerged very early in animal evolution. In fact, the common ancestor of cnidarians, protostomes and deuterostomes already contained a set of RBR proteins which was almost identical to the one found today in humans. Genes of 10 subfamilies were present in that ancestor. Since then, and surprisingly, many animal lineages have lost RBR genes. For example, just six genes are present in the fruit fly *Drosophila melanogaster*. Microarray data indicated that conservation is linked to the proteins having housekeeping functions, while more specialized genes tend to be lost [11]. The significance of that study was to provide a conceptual framework for the reasons that explain the long-term pattern of conservation and loss of the genes of the RBR family. A significant difference in the process of conservation/loss in other groups would be an evidence for a modification in the functions of the RBR proteins respect to those found in animals.

Plant RBRs have not been hitherto studied as a whole, but the available data suggest that the evolutionary dynamics of this family in plants may be very different from that described in animals. First, it was shown that *Arabidopsis thaliana* contained many more RBR genes than any animal, and about three times more than humans [8]. Second, although part of the diversity of *Arabidopsis* RBRs certainly may have emerged as a consequence of the genome duplications that occurred in its lineage [12]–[15], analyses of the members of one of the RBR subfamilies, called Ariadne, showed that many of them actually arose by recent tandem duplications [16]. Finally, the number of subfamilies found in plants was limited to four, and just one of them was determined to be plant-specific [5], [8], [10]. All these results together suggest that the evolutionary mechanisms governing the patterns of diversification of plant and animal RBR may be somewhat contradictory. Given these preliminary data and the raising interest in the evolution and function of the ubiquitination system in plants (e. g. refs. [17]–[21] and see also [22], [23]), I have decided to perform a comprehensive analysis of plant RBRs in order to obtain a better understanding of the evolution and function of these proteins. The study highlights some basic similarities in the evolution of plant and animal RBRs, but also demonstrates fundamental differences that may be linked to qualitatively different functional roles of these proteins in the two groups of organisms.

## Results

### General patterns of diversification of plant RBR ubiquitin ligases

**Figure 1:**
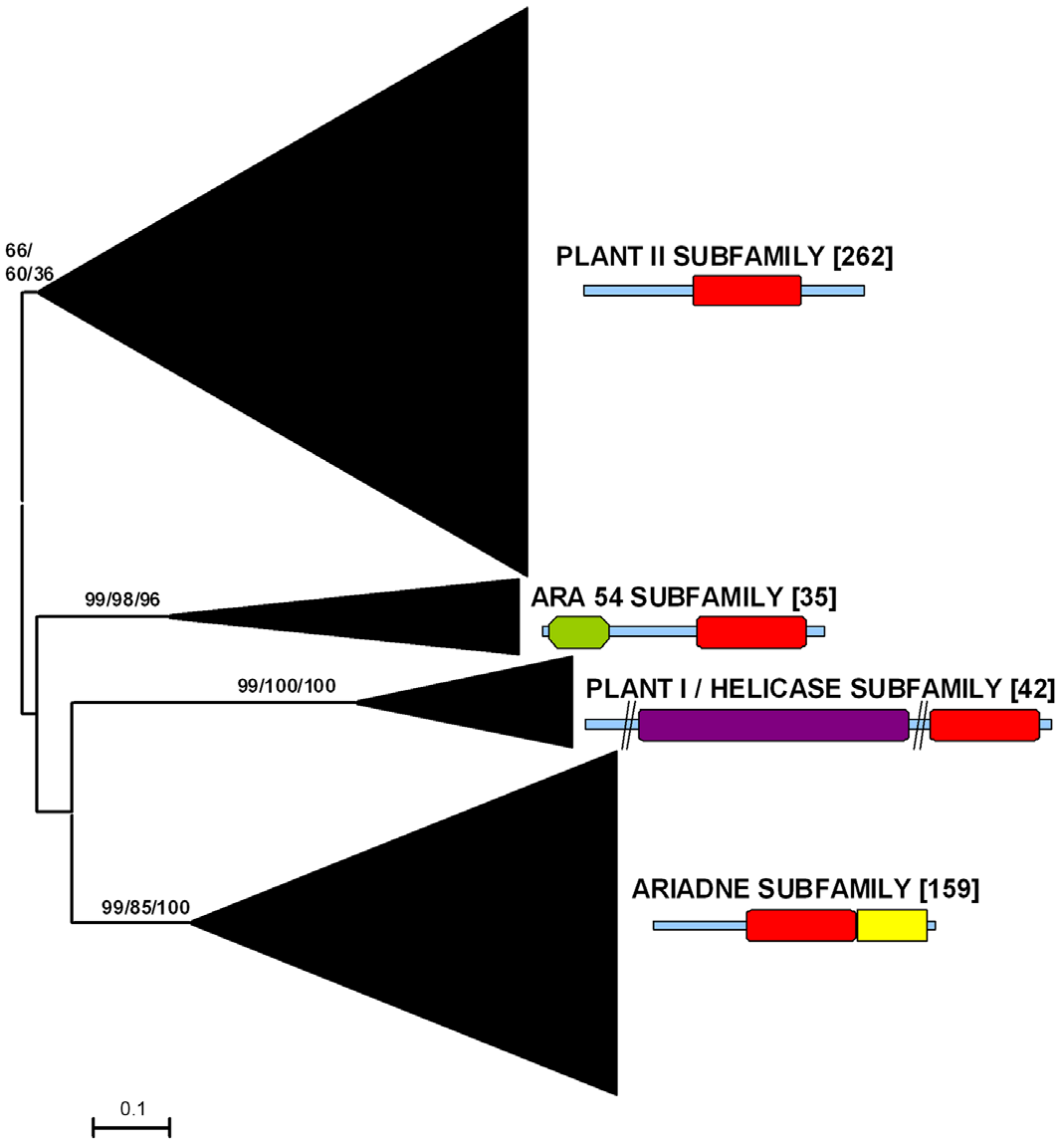
Basic result for the general analyses including 498 plant RBR sequences. The main branches that correspond to the four subfamilies are indicated. Numbers above those branches correspond to bootstrap support, in percentages. The three numbers correspond to Neighbor-joining (NJ), maximum parsimony (MP) and maximum likelihood (ML) analyses (order: NJ/MP/ML). Numbers in brackets refer to the number of protein sequences which are included in a branch. The typical structures of the proteins in the subfamilies are also indicated (red: RBR supradomain; green: RWD/GI domain; purple: DEAD/DEAH helicase domain; yellow: Ariadne domain). The slashes in the Helicase-containing protein are included to reflect that these proteins are usually much longer than shown here and two regions have been deleted. See ref. [5] for further details of these structures.

Previous results using small datasets suggested that all plant RBR proteins could be classified into just four subfamilies [5], [8], [10]. Three of them (Ariadne, ARA54 and Plant I/Helicase) are ancient, given that can be found in both unikonts and bikonts [8], [11]. The fourth family (called Plant II) was found only in higher plants. Figure 1 summarizes the general results obtained using a much larger dataset, consisting in 498 plant sequences (see Material and Methods). Confirming those previous results, all sequences could be classified into the four mentioned subfamilies using phylogenetic analysis and structural data. The low bootstrap values supporting the Plant II subfamily are due both to the intrinsic high heterogeneity of the sequences of this group and to the presence of a particularly divergent sequence from the green alga *Micromonas pusilla* (Accession number ACCP01000105.1). If this sequence is eliminated, bootstrap support for the Plant II subfamily is much higher (e. g. 92% of support in neighbor-joining analyses). Extensive structural searches using InterProScan also confirmed previous results: all the proteins of the ARA54, Ariadne and Helicase subfamilies tested had not only the RBR supradomain, but also additional, characteristic protein domains (summarized in [5]; see Figure 1), which are absent in Plant II subfamily proteins. Structural data further supports the inclusion of the divergent *M. pusilla* sequence into the Plant II subfamily, given that no additional protein domains were detected in it.

The genome projects of species that belong to three different genera of green algae (*Chlamydomonas*, *Ostreococcus* and *Micromonas*) have provided a number of RBR sequences which were not previously available and are obviously critical to understand the early evolution of this family in plants. All those sequences belong to three of the four subfamilies, namely Plant II, ARA54 and Ariadne. Significantly, in the available genomes not only of green algae, but also of bryophytes and gymnosperms proteins of the ancient helicase subfamily were not found. They have been detected so far only in angiosperms. A similar loss has been observed in multiple animal lineages, suggesting that helicase RBRs are often dispensable [11]. However, a significant caveat is that 91% (452/498) of the available sequences derive from angiosperm genomes. Therefore, to find in the future helicase RBRs in other groups would not be surprising. In any case, these results demonstrate that the four subfamilies found so far in plants originated before the split that separated green algae from the rest of plants. This indicates that at least four RBR genes were present in the common ancestor of all viridiplantae. The fact that no additional subfamilies have appeared along the evolution of the plant lineages contrasts with the pattern found in animals, in which many additional subfamilies have emerged [11].

**Figure 2 pone-0011579-g002:**
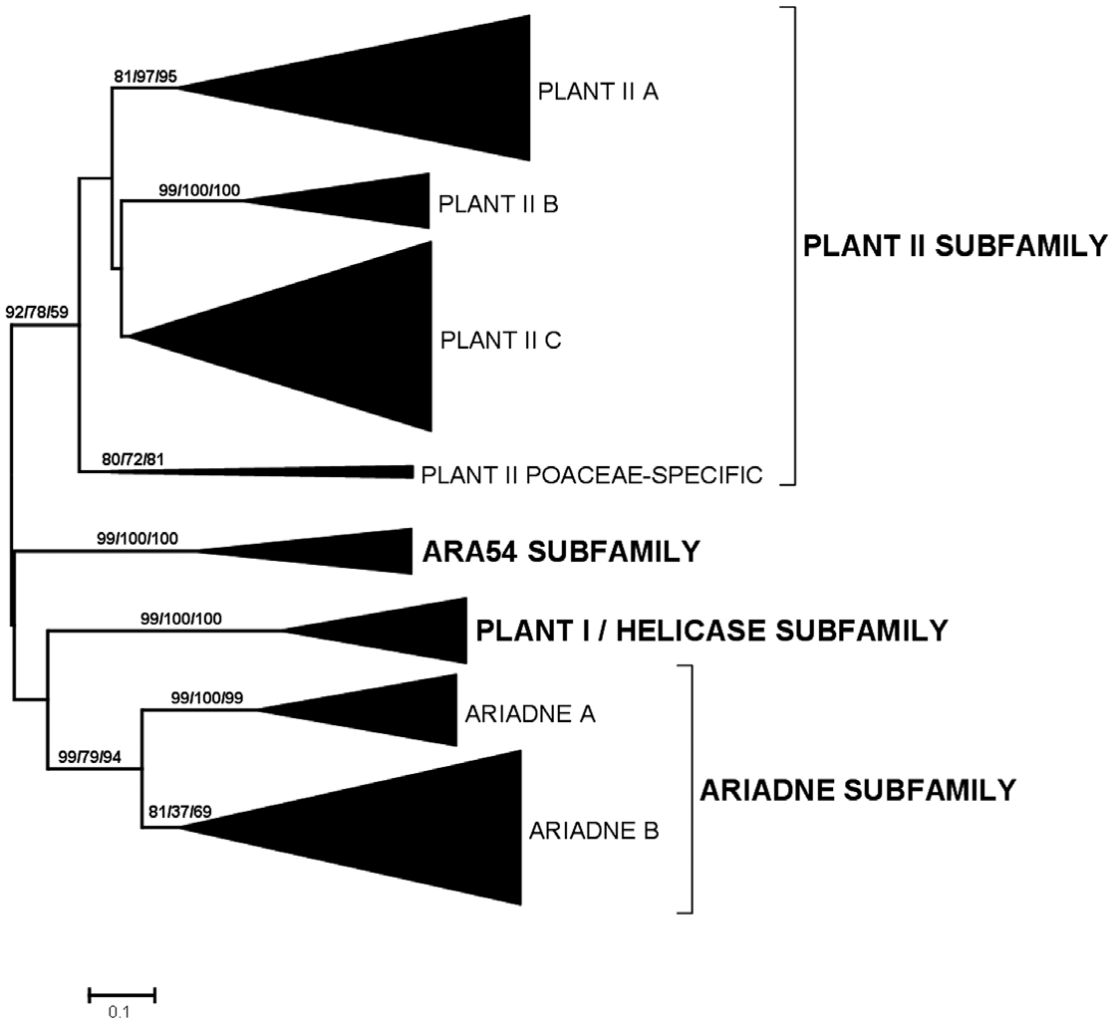
Dendrograms for RBR sequences of angiosperms and gymnosperms. Bootstrap support and number of sequences indicated as in Figure 1. Bootstrap values (in percentages) for highly supported branches are indicated.

Figure 2 summarizes the phylogenetic analyses for the dataset of 472 sequences detected in angiosperms and gymnosperms. This analysis was performed in order to explore in detail the diversification of the RBR subfamilies in these groups. Interestingly, two highly supported monophyletic groups were detected within the Ariadne subfamily and three within the Plant II subfamily (Figure 2). A fourth potential group of the Plant II subfamily, which is indicated as “Plant II C” in Figure 2, is in fact a bit of a ragbag, given that there is no significant bootstrap support for it. It just includes all the sequences in the Plant II subfamily which are excluded from the highly supported A, B and Poaceae-specific groups. However, as it will be shown below, the Plant II C group indeed appears as strongly supported in other analyses, so it seems useful for operative reasons to define it at this point of the study. Sequences of the Ariadne B, Plant II A, Plant II B and Plant II C groups were detected in both angiosperms and gymnosperms. On the contrary, the Plant II Poaceae-specific group was found, as the name indicates, only in a few species of that family of monocot plants. Also, Ariadne A sequences were not detected in gymnosperms. This means that there were no less than 6 RBR genes before the gymnosperm/angiosperm split (at least 3 Plant II genes, single ARA54 and Helicase genes and an Ariadne gene). In fact, it can be deduced that the number of genes in the common ancestor of angiosperms and gymnosperms was at least 7 and probably no less than 8. This is due to two factors. First, the heterogeneous nature of the Plant II C group, already mentioned. Inspection of that group indicated that it contains two separated sets of sequences in gymnosperms and several in angiosperms. This means that the common ancestor of those two groups of plants may have had at least 2 Plant II C genes. Second, it turns out that a green alga, *Chlamydomonas reinhardtii*, contains two Ariadne proteins that are very similar to, respectively, Ariadne A and Ariadne B sequences (this fact, strongly supported by bootstrap data, was observed in the analyses from which Figure 1 was obtained). This result indicates that the two Ariadne groups emerged very early in plant evolution and were therefore already present before angiosperms and gymnosperms diverged. The lack of Ariadne A genes in gymnosperms, if indeed confirmed when more sequences are available and not simply due to a lack of information for gymnosperm genomes, must be therefore be attributed to a subsequent loss. Interestingly, this finding of two Ariadnes in *Chlamydomonas* also rises the number of genes deduced for the common ancestor of all plants to 5, instead of the four indicated above.

Table 1, which summarizes the number of RBR genes in selected plant genomes, adds interesting information to understand the early patterns of diversification of RBR genes in plants. By carefully inspecting the results summarized in Figures 1 and 2, almost all the sequences of these species were assigned without ambiguity to one of the groups already mentioned. The only exceptions were 7 sequences from green algae or the bryophyte *Physcomitrella patens*, which were impossible to classify. Five of those 7 sequences (four from *Physcomitrella* and the highly divergent sequence from the green alga *Micromonas pusilla* already mentioned before) belong to the Plant II subfamily, but were not clearly assignable to any of the Plant II groups defined in Figure 2. The other two sequences (from green algae of the *Ostreococcus* genus) were highly divergent Ariadne sequences that again could not be assigned to either the Ariadne A or Ariadne B groups. The fact that sequences belonging to the Plant II groups (A, B and C) defined above cannot be described in either green algae or bryophytes is compatible with the idea that these three groups emerged just before the angiosperm/gymnosperm split.

**Table 1:** Number of RBR sequences in selected species.

SPECIES	Taxonomic group	PLANT II A	PLANT II B	PLANT II C	PLANT II poaceae-specific	PLANT II Others	ARA54	HELICASE	ARIADNE A	ARIADNE B	ARIADNE Others	TOTAL
***Chlamydomonas reinhardtii***	Green algae	0	0	0	0	0	1	0	1	1	0	3
***Ostreococcus lucimarinus***	Green algae	0	0	0	0	0	1	0	0	0	1	2
***Ostreococcus tauri***	Green algae	0	0	0	0	0	1	0	0	0	1	2
***Micromonas pusilla***	Green algae	0	0	0	0	1	1	0	0	1	0	3
***Physcomitrella patens***	Bryophytes	0	0	0	0	4	1	0	2	4	0	11
***Picea glauca***	Gymnosperms	0	1	4	0	0	0	0	0	1	0	6
***Pinus taeda***	Gymnosperms	0	0	6	0	0	1	0	0	0	0	7
***Zea mays***	Angiosperms, monocots	1	1	4	0	0	1	1	2	4	0	14
***Sorghum bicolor***	Angiosperms, monocots	1	0	4	3	0	4	1	3	8	0	24
***Oryza sativa***	Angiosperms, monocots	1	1	6	2	0	6	1	4	5	0	26
***Solanum lycopersicon***	Angiosperms, dicots, asterids	0	1	1	0	0	1	1	2	2	0	8
***Nicotiana tabacum***	Angiosperms, dicots, asterids	0	1	4	0	0	0	2	2	3	0	12
***Mimulus guttatus***	Angiosperms, dicots, asterids	1	1	0	0	0	0	0	1	1	0	4
***Vitis vinifera***	Angiosperms, dicots, rosids	1	1	3	0	0	1	2	2	2	0	12
***Glycine max***	Angiosperms, dicots, rosids	2	2	6	0	0	0	1	0	1	0	12
***Lotus japonicus***	Angiosperms, dicots, rosids	1	1	6	0	0	0	0	0	2	0	10
***Medicago truncatula***	Angiosperms, dicots, rosids	1	1	4	0	0	0	2	0	4	0	12
***Populus trichocarpa***	Angiosperms, dicots, rosids	2	2	5	0	0	1	1	1	6	0	18
***Ricinus communis***	Angiosperms, dicots, rosids	1	1	5	0	0	1	1	2	3	0	14
***Arabidopsis thaliana***	Angiosperms, dicots, rosids	18	1	3	0	0	1	3	4	12	0	42
***Arabidopsis lyrata***	Angiosperms, dicots, rosids	18	1	3	0	0	1	1	4	2	0	30
***Brassica olearacea***	Angiosperms, dicots, rosids	5	1	0	0	0	0	2	0	2	0	10
***Brassica rapa***	Angiosperms, dicots, rosids	5	1	2	0	0	1	2	3	3	0	17
***Carica papaya***	Angiosperms, dicots, rosids	3	1	2	0	0	0	1	0	3	0	10

Table 1 contains additional interesting information. First, all the green algae sequenced so far contain 2–3 RBR genes, when the common ancestor of all plants had at least 5, as already indicated. Therefore, a few lineage-specific losses have occurred. Second, the only bryophyte for which a significant amount of data is available, *Physcomitrella patens*, contains a relatively large number of RBR genes, bearing lineage-specific duplications of Plant II and Ariadne RBRs, but, as already indicated above, lacking helicase RBRs. Third, the gymnosperms for which the most data are available seem to contain a limited number of genes (6–7 per species) while angiosperms usually contain quite more (often 10–14). Although this result is interesting, whether this difference is real or just it is due to a lack of data for gymnosperm species is still unclear. Finally, some angiosperm species, especially the monocots *Sorghum bicolor* and *Oryza sativa* and the dicots of the *Arabidopsis* genus (*A. thaliana* and *A. lyrata*) contain a very large amount of RBR proteins (25–40). This last result, which implies at least two independent lineage-specific patterns of amplification, may be studied in more detail, given the large number of sequences available from the families Poaceae and Brassicaceae, to which those species belong (see next section).

**Figure 3 pone-0011579-g003:**
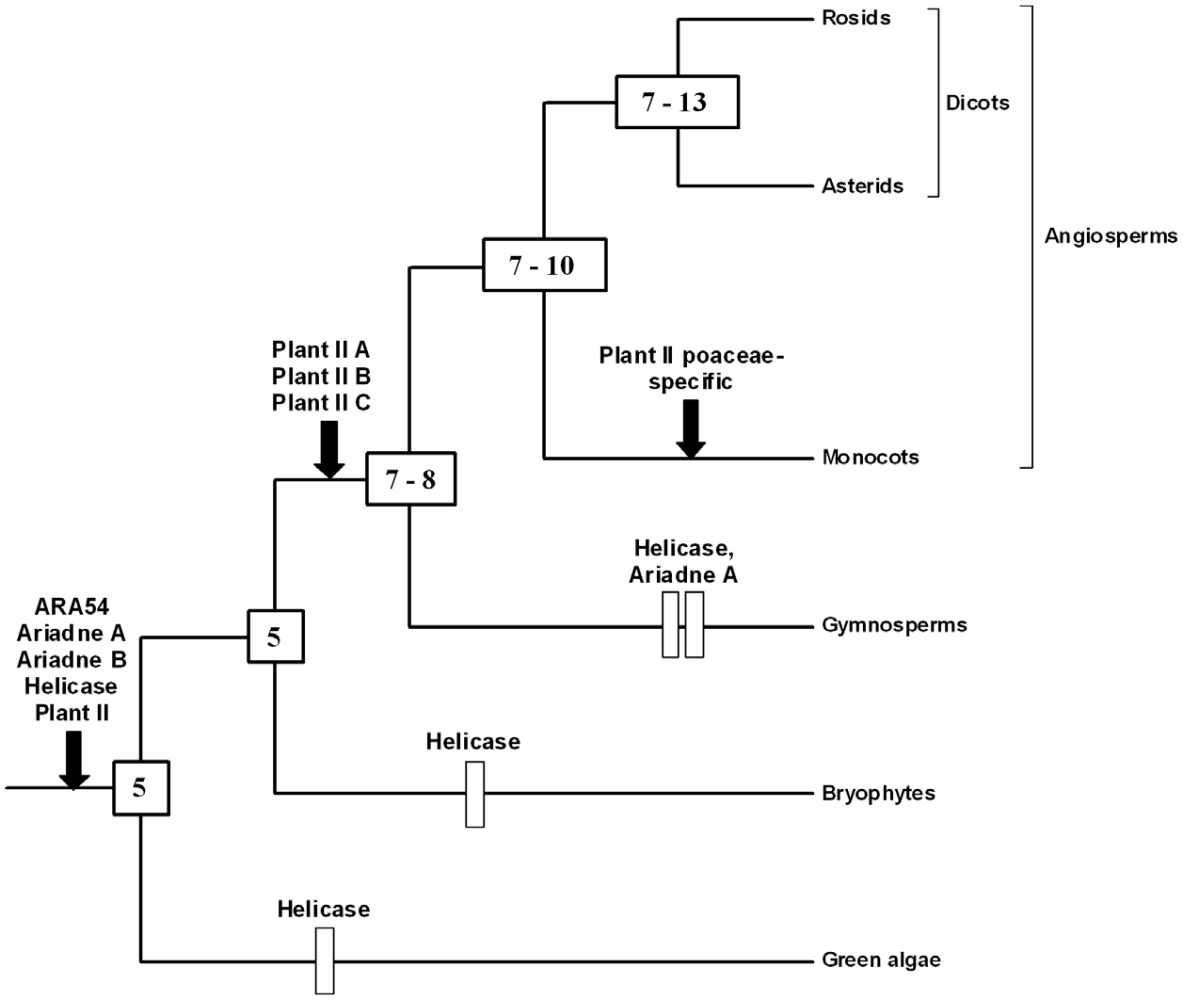
The most parsimonious hypothesis to explain the progressive diversification of plant RBR genes. This figure summarizes how this family may have diversified, according to the available data. Rectangles correspond to gene losses and arrows to gene emergences. The numbers in the internal nodes (boxes) correspond to the genes deduced to exist at that particular time. Ambiguities are due to the difficulties in establishing when lineage-specific duplications occurred (see text).

In Figure 3, I propose a hypothesis for the diversification of the RBR family in plant lineages that summarizes all the currently available data. This figure has been obtained by exploring in detail the branching patterns found in the analyses shown in Figure 1 and 2 and looking for the most parsimonious explanations for those patterns. Hypothesizing gene gains is more parsimonious than assuming that gains plus subsequent losses have occurred. Therefore, this hypothesis is based on minimizing the number of gene losses that must be postulated to explain the genes observed, what may lead in some cases to an underestimation of the true number of ancestral genes. For those estimations, the minimum number of genes for an ancestor was considered equal to the number of monophyletic groups, strongly supported by bootstrap analyses, present in all their descendant lineages. The maximum number was appraised by establishing the number of genes in each descendant lineage (excluding very recent duplicates) and assuming that the number of ancestral genes is equal to the number of genes present in the descendant lineage which has less of them. This method will have a tendency to produce overestimates of the number of genes, given that it often assumes that genes in different lineages are orthologs even when evidence for this orthology is absent. It is therefore mainly useful to obtain a top estimate of the genes present in an ancestor. The summary of Figure 3 is that, as already deduced above, 5 genes must be postulated at the origin of plants. Afterwards, the most parsimonious hypothesis involves a slow increase of the number of genes along plant evolution. Sporadic losses have also apparently occurred, but, contrary to what has been described in animals [11], particular lineages that have suffered massive losses of RBR genes have not been found in plants. Although this hypothesis should be revised when more data are available, the current information is quite large, so the general pattern is robust.

### Microevolutionary patterns in the Poaceae and Brassicaceae families

**Figure 4 pone-0011579-g004:**
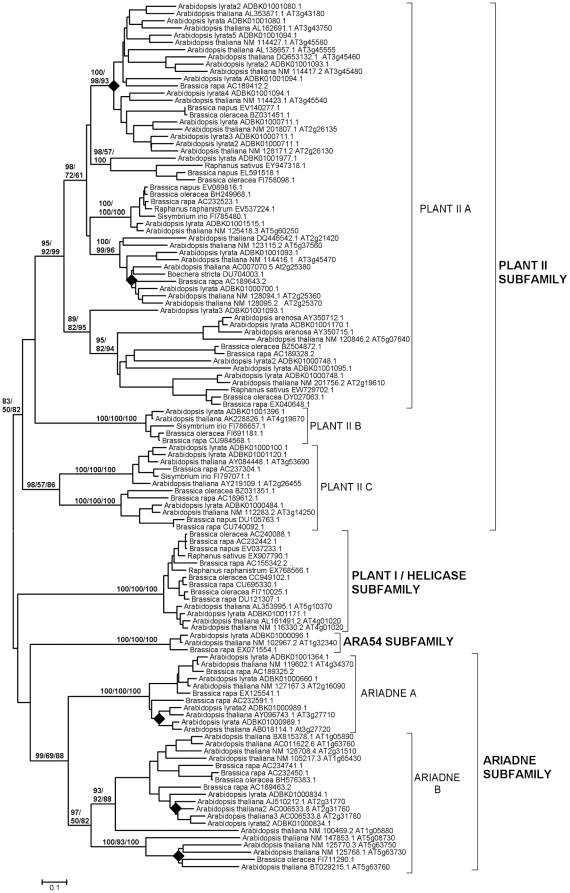
Dendrogram for Brassicaceae RBRs. Rhombs indicate the branches that contain tandem duplicates in *Arabidopsis thaliana*. Numbers refer again to bootstrap support, in percentage (NJ/MP/ML). For simplification, support for external branches has not been included.

**Figure 5:**
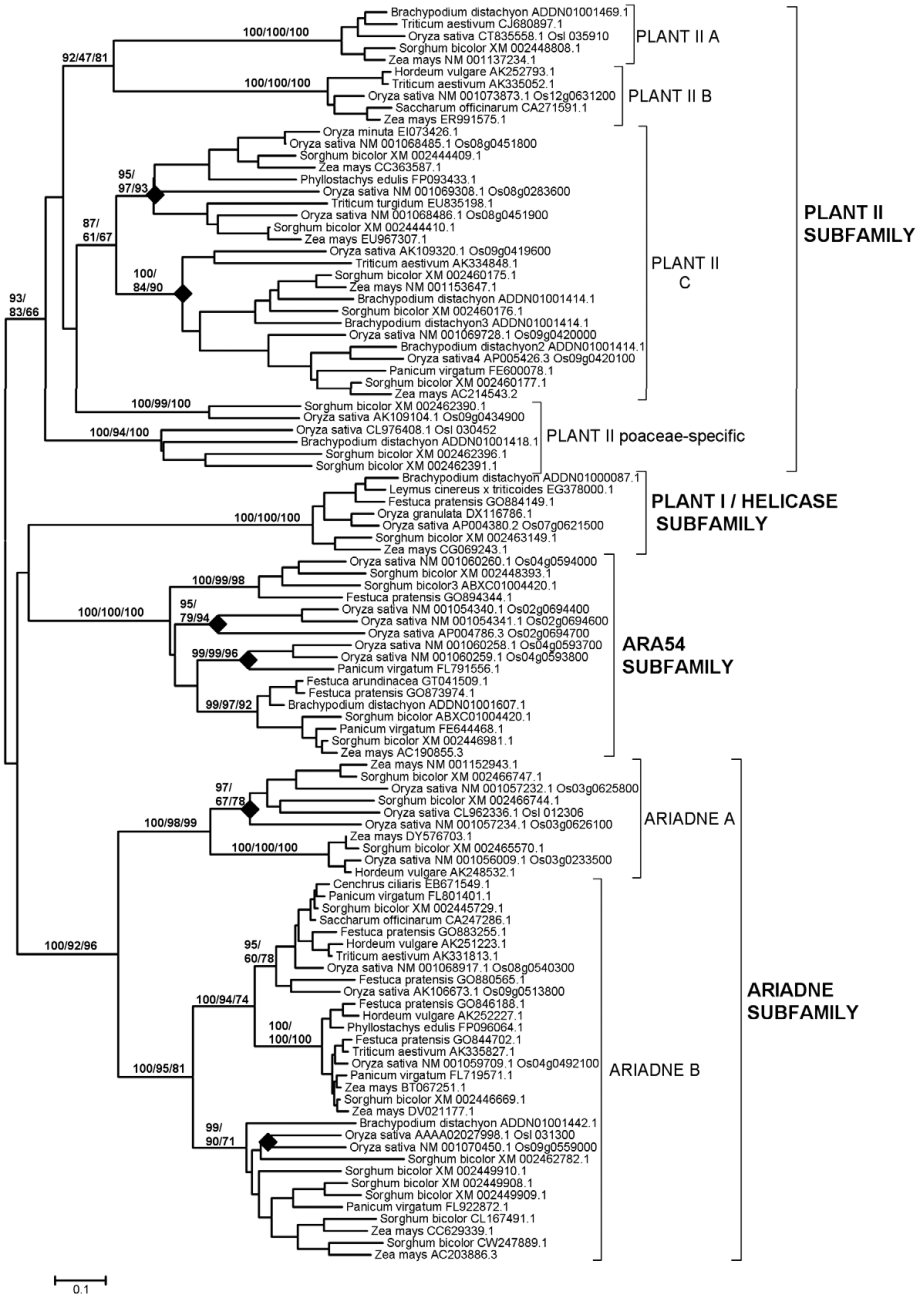
Dendrogram for Poaceae RBRs. Conventions as in Figure 4. Again, rhombs indicate the branches that include tandem duplicates, this time in *Oryza sativa*.

In this section, the contribution of gene-specific duplications to explain the current number of RBR genes in two well-known lineages, the family Brassicaceae (Cruciferae), today the best-studied dicot group, and the family Poaceae (Gramineae), the group for which almost all available monocot sequences derive, is examined. Figures 4 and 5 summarize the dendrograms obtained when sequences from, respectively, Brassicaceae and Poaceae were analyzed. In general, they confirmed the findings already described in the previous section. It is particularly significant to point out the high support found in these analyses for the Plant II C group defined above. In addition, these more concrete analyses allow to establish the presence of several highly supported branches within the groups Plant II A, Plant II C and Ariadne B of the Brassicaceae (Figure 4) and Plant II C, ARA54, Ariadne A and Ariadne B in the Poaceae (Figure 5). In summary, it can be deduced that the total number of genes in the ancestor of all Brassicaceae was about 16 and approximately 12 genes were present in the Poaceae ancestor. It is reasonable to hypothesize that many of these genes originated from the well-documented, ancient genomic duplications that occurred in those lineages. In addition, Figures 4 and 5 also include analyses for tandem duplications in *Arabidopsis thaliana* and *Oryza sativa*. The rhombs in those figures mark branches that include two or more tandemly repeated genes, as can be deduced from their genomic locations. I confirmed the presence of tandemly repeated Ariadnes (of both groups, A and B) in *Arabidopsis*, as first described in [16], and also found multiple tandem duplicates in the Plant II A group in that same species (Figure 4). These genes are not found in the species of the evolutionary close *Brassica* genus. Multiple genus-specific duplicates were also found in *Oryza* (Figure 5). Their pattern was clearly distinct from the one found in *Arabidopsis*, with rice duplicates belonging to the Plant II C, Ariadne A and Ariadne B groups and to the ARA54 subfamily. These results not only demonstrate that the tandem duplications arose recently and independently in *Arabidopsis* and *Oryza*, but also that each species has a preferential pattern of tandem duplications.

### Patterns of expression of RBR genes in *Arabidopsis* and *Oryza*

A significant point that arose when analyzing animal RBR genes was the correlation found between their evolutionary conservation and their patterns of expression. Genes with broad patterns of expression, probably those that have housekeeping functions, were preferentially conserved, while more specialized genes were often lost [11]. Given this precedent, the correlation between pattern of expression and evolutionary conservation and emergence of duplicates in two model plant species, *Arabidopsis thaliana* and *Oryza sativa*, was analyzed. First, the database generated by Schmid et al. [24], which contains expression data obtained using microarrays for 79 developmental stages of *Arabidopsis thaliana*, was used. AtGenExpress, in which those data are deposited, contained information about 31 of the *Arabidopsis* RBR genes (data obtained from http://jsp.weigelworld.org/expviz/expviz.jsp; see Material and Methods). It turned out that these 31 genes could be classified into two different classes, based on quantitative differences in their expression patterns. Eleven genes had average levels of expression ranging from 42.6 units to 545.0 units, with an average of 190.3±41.6 units. The rest had a very low average level of expression (11.3±1.0 units; range; 5.5–16.2). Given that these groups were established *a posteriori*, to test whether this difference in expression between both classes was relevant, Hochberg's GT2 method for unplanned comparisons was used (see Materials and Methods). The difference was found to be statistically significant (p<0.01). It was also observed that the low expressed genes had a mean level of expression in mature pollen (mean: 52.5±9.5) that was in average about 5 times higher than the mean for the rest of samples (10.7±1.0). This difference is also significant (p<0.01; again using the GT2 method). Interestingly, all the genes that were included in the clusters of tandem duplicates (rhombs in Figure 4) for which expression data was available, a total of 13, were included in the group of low-level, specifically expressed genes.

**Figure 6:**
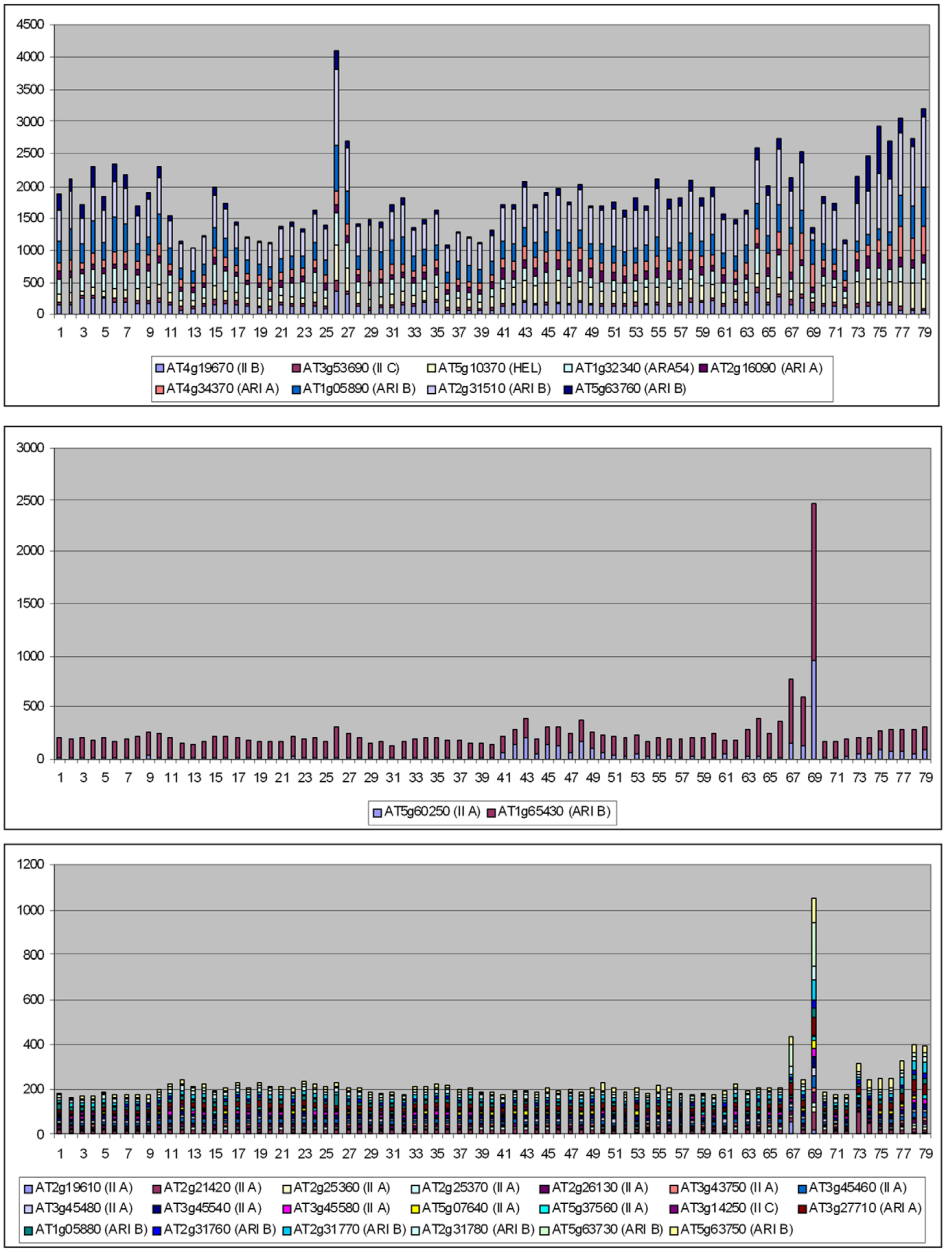
Cumulative values of expression for *Arabidopsis* RBR genes in 79 developmental samples. Data from Schmid et al. [24]. Top: broadly expressed RBR genes, notice the high average levels of expression in all tissues. Center: two genes show high levels of expression but especially in one tissue (Mature pollen: sample 69). Bottom: set of genes expressed at very low level in all tissues with the exception of mature pollen. Samples were as follows: 1) root 7 days; 2) root 17 days; 3) root 15 days; 4) root 8 days; 5) root 8 days; 6) root 21 days; 7) root 21 days; 8) stem: hypocotyl; 9) stem: first node; 10) stem: second internode; 11) cotyledons; 12) leaves 1+2; 13) rosette leaf #4, 1 cm long; 14) rosette leaf #4, 1 cm long (gl1-T mutant); 15) rosette leaf #2; 16) rosette leaf #4; 17) rosette leaf #6; 18) rosette leaf #8; 19) rosette leaf #10; 20) rosette leaf #12; 21) rosette leaf #12 (gl1-T mutant); 22) leaf 7, petiole; 23) leaf 7, petiole; 24) leaf 7, distal half; 25) leaf, 15 days; 26) leaf, senescing; 27) cauline leaves; 28) seedling, green parts, 7 days; 29) seedling, green parts, 8 days; 30) seedling, green parts, 8 days; 31) seedling, green parts, 21 days; 32) seedling, green parts, 21 days; 33) whole plant: developmental drift, entire rosette after transition to flowering, but before bolting, 21 days; 34) whole plant: developmental drift, entire rosette after transition to flowering, but before bolting, 22 days; 35) whole plant: developmental drift, entire rosette after transition to flowering, but before bolting, 23 days; 36) vegetative rosette 7 days; 37) vegetative rosette 14 days; 38) vegetative rosette 21 days; 39) shoot apex, vegetative + young leaves; 40) shoot apex, vegetative; 41) shoot apex, transition (before bolting); 42) shoot apex, inflorescence (after bolting); 43) shoot apex, inflorescence (after bolting) (clv3-7 mutant); 44) shoot apex, inflorescence (after bolting) (lfy-12 mutant); 45) shoot apex, inflorescence (after bolting) (ap1-15 mutant); 46) shoot apex, inflorescence (after bolting) (ap2-6 mutant); 47) shoot apex, inflorescence (after bolting) (ufo-1 mutant); 48) shoot apex, inflorescence (after bolting) (ap3-6 mutant); 49) shoot apex, inflorescence (after bolting) (ag-12 mutant); 50) flowers stage 9; 51) flowers stage 10/11; 52) flowers stage 12; 53) flower stage 12; multi-carpel gynoeceum; enlarged meristem; increased organ number (clv3-7 mutant); 54) flower stage 12; shoot characteristics; most organs leaf-like (lfy-12 mutant); 55) flower stage 12; sepals replaced by leaf-like organs, petals mostly lacking, has secondary flowers (ap1-15 mutant); 56) flower stage 12; no sepals or petals (ap2-6 mutant); 57) flower stage 12; filamentous organs in whorls two and three (ufo-1 mutant); 58) flower stage 12; no petals or stamens (ap3-6 mutant) 59) flower stage 12; no stamens or carpels (ag-12 mutant); 60) flowers stage 15; 61) flowers 28 days; 62) flowers stage 15, pedicels; 63) flowers stage 12, sepals; 64) flowers stage 15, sepals; 65) flowers stage 12, petals; 66) flowers stage 15, petals; 67) flowers stage 12, stamens; 68) flowers stage 15, stamen; 69) mature pollen 70) flowers stage 12, carpels; 71) flowers stage 15, carpels; 72) siliques, w/ seeds stage 3; mid globular to early heart embryos; 73) siliques, w/ seeds stage 4; early to late heart embryos; 74) siliques, w/ seeds stage 5; late heart to mid torpedo embryos; 75) seeds, stage 6, w/o siliques; mid to late torpedo embryos; 76) seeds, stage 7, w/o siliques; late torpedo to early walking-stick embryos; 77) seeds, stage 8, w/o siliques; walking-stick to early curled cotyledons embryos; 78) seeds, stage 9, w/o siliques; curled cotyledons to early green cotyledons embryos; 79) seeds, stage 10, w/o siliques; green cotyledons embryos. See details in [24].

Figure 6 graphically summarizes these results, adding some details. In the top panel, the expression in all samples of nine of the highly expressed genes is shown. In the middle panel, the other two genes of this group (*AT5g60250* and *AT1g65430*) are singled out to show that they have a particularly high level of expression in just one developmental sample, which is again mature pollen (8 times the average of the rest of samples for *AT5g60250* and 23 times for *AT1g65430*). This specificity was not observed in any of the other highly expressed genes. For example, in the nine genes shown in the top panel, the differences between the sample with the highest level of expression and the sample with the second highest level was always very small (1.2±0.2 times; range 1.1–1.6). However, expression in mature pollen was 2.5 times (*AT5g60250*) or 4.7 times (*AT1g65430*) higher than the expression of the second highest developmental sample for these two genes. Not surprisingly, they both were also detected as expressed at a higher level in pollen than in vegetative tissues in an independent dataset [25]. Finally, the bottom panel of Figure 6 shows the patterns of the genes with low levels of expression. When all expression level values are added together, the high level of the pollen samples becomes easily visible.

**Figure 7:**
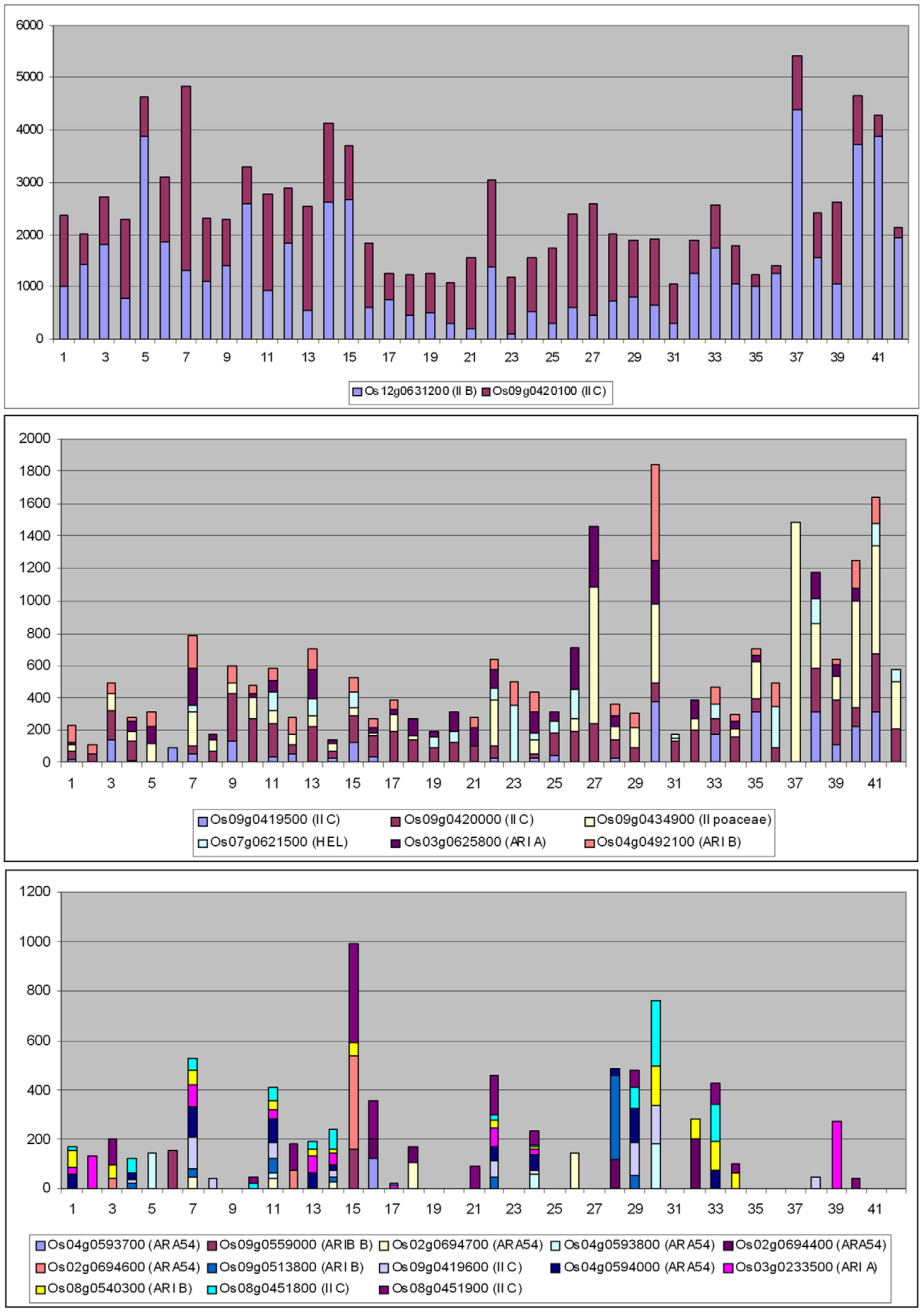
Cumulative values of expression for *Oryza* RBR genes in 42 cell types. Data from [26]. Top: broadly expressed RBR genes with high average levels of expression in all cell types. Center: genes also broadly expressed, but at lower levels. Bottom: set of genes expressed at very low levels in most tissues. The cell types from which the data derive are as follows: 1) Scutellum (0 hr); 2) Scutellum (12 hr); 3) Scutellum (24 hr); 4) Coleoptile (0 hr); 5) Coleoptile (12 hr); 6) Coleoptile (24 hr); 7) Plumule (0 hr); 8) Plumule (12 hr); 9) Plumule (24 hr); 10) Epiblast (0 hr); 11) Epiblast (12 hr); 12) Epiblast (24 hr); 13) Radicle (0 hr); 14) Radicle (12 hr); 15) Radicle (24 hr); 16) Axillary primordium; 17) Axillary meristem 18) Apical meristem; 19) P1; 20) P2; 21) P3; 22) Seedling blade bulliform; 23) Seedling blade stomata; 24) Seedling blade long cell; 25) Seedling blade mesophyll; 26) Seedling blade bundle sheath; 27) Seedling blade vein; 28) Lateral root cap; 29) Root tip cortex; 30) Root tip vascular bundle; 31) Root tip metaxylem; 32) Elongation epidermis; 33) Elongation cortex; 34) Elongation endodermis; 35) Elongation vascular bundle; 36) Elongation metaxylem; 37) Maturation epidermis; 38) Maturation cortex; 39) Maturation endodermis; 40) Matur. vascular bundle; 41) Whole root; 42) Whole leaf (fresh). Details can be found in [26].

Data from *Oryza sativa japonica* were obtained from the RiceAtlas website (http://bioinformatics.med.yale.edu/riceatlas/; [26]), which contains information about 42 different cell types. The information available for 21 *Oryza* genes was obtained and it was determined that they were also divisible into three groups, which I will call respectively Class I, Class II and Class III, according to both their average level of expression and breadth of expression (summarized in Figure 7). Class I includes just two genes (*Os09g0420100* and *Os12g0631200*) with a very high level of expression in all cell types (Average levels: 1106.3±92.9 and 1366.2±43.9 respectively; Figure 7, top panel). Class II includes 6 genes that were also expressed in many cell types (an average of 27.8 of the 42 samples, ranging from 17 to 38 for the different genes) but at a lower level (Averages of expression level ranging from 45.4±11.8 to 170.0±43.9; Figure 7, middle). Finally, Class III includes the other 13 genes, which were expressed very specifically (just 7.3 cell types in average; range: 1–13), and generally at low levels (Average expression level ranging from 2.8±2.8 to 31.7±11.1; Figure 7, bottom). As happened in *Arabidopsis*, out of the 14 genes found in tandem duplicate sets for which information was available, 10 were included in the group of low-expressed genes. It is significant that no pollen-derived sample was included in the *Oryza* dataset, so no further comparisons with the *Arabidopsis* data are possible. The quantitative differences in the levels of expression between the three groups defined above were all significant (p<0.01 for Class I vs. Class II and Class I vs. Class III comparisons and 0.01<p<0.05 for the Class II vs. Class III comparison; in all cases, probabilities were established using the GT2 test).

Some additional functional information for *Arabidopsis* RBR genes exists. In particular, data at AtGenExpress, the databases at The Arabidopsis Information Resource (TAIR; http://www.arabidopsis.org/ [27]) and the Gene expression omnibus (GEO; http://www.ncbi.nlm.nih.gov/geo/) were checked for potential information about the conditions that may trigger the expression of RBR genes. Analyses using the AtGenExpress Visualization Tool failed to detect a consistent pattern for external conditions that may lead to activation of the pollen-specific, low-expression genes detected in *Arabidopsis*. It was just detected an induction of expression of a few of them under certain abiotic or biotic stress conditions (*AT1g05880*: *Pseudomonas* infection, cold stress, salt stress, genotoxic stress caused by bleomycin and mytomycin C, UV-induced stress, heat; *AT5g63750*: heat stress in cultured cells; *AT3g27710*: heat stress in cultured cells). However, none of these experiments was performed using pollen samples, so the results can be considered inconclusive. Examination of the data deposited at the Gene expression omnibus (GEO; http://www.ncbi.nlm.nih.gov/geo/) also failed to show any consistent pattern (not shown). More interesting were the results obtained from TAIR. All the conditions indicated in the TAIR databases as to having an effect on RBR gene expression levels were tabulated and a significant regularity was detected. Five of the 9 highly expressed genes of *Arabidopsis* (Figure 6, top panel) were found to be overexpressed after infecting the plants with the cabbage leaf curl virus, a geminivirus [28]. Given that only 13.3% (3004/22500) of the probes analyzed were overexpressed in those experiments, this enrichment is highly significant (p = 0.0012; Chi square test with Yates correction, 1 degree of freedom).

## Discussion

The amount of sequence information for plant RBRs is quite heterogeneous, with some lineages (e. g. angiosperms) very well represented, while information for other groups is quite limited. This makes difficult to establish without ambiguity the patterns of differentiation of this family of proteins. Even with this caveat in mind, several general conclusions can be safely deduced. First, the available data are fully compatible with the idea that while green algae have kept a set of RBR genes a bit smaller than the one that existed when viridiplantae originated, a significant increase in the number of these genes has occurred in the lineages that gave rise to higher plants and, particularly, large increases in angiosperm lineages are common (Figure 3 and Table 1). Appearance of new genes of the four subfamilies has been the rule, while losses have been, in general, rare. Exceptional are the helicase RBR genes, which, according to the available data (summarized in Figure 3), may have been lost independently in chlorophyta, bryophyta and gymnosperms. As already mentioned, this loss of helicase genes, if confirmed when more information for these groups is available, would mirror the multiple independent losses of those same genes in animal lineages [11]. Second, no new types of proteins have emerged since the origin of plants. This leads to all analyzed plants having a set of RBR ubiquitin ligases which is structurally very similar or even identical. We can conclude that the RBR family has followed in plants a pattern of microdifferentiation in which most of the variation is in the number of genes, while the types of proteins generated remain basically the same present at the origin of the viridiplantae. Third, as mentioned above, part of the progressive diversification of RBR genes most likely has been associated to the multiple whole genome duplications that occurred in higher plant lineages. However, the relatively slow increase in the total number of genes in all lineages (Figure 3) suggests that RBR genes are, as a whole, quite “resistant” to genome duplications, that is, most genes produced after these duplications tend to be lost [29]. Contrasting with this long-range difficulty of accommodating additional genes, it turns out that gene-specific duplications, which lead to tandemly repeated genes, explain many of the genes found today in the species with the highest number of RBR genes (such as *Arabidopsis thaliana* or *Oryza sativa*; Figures 4 and 5).

The patterns of diversification of the RBR family in plants and in animals seem quite different. Contrary to what happened in plants, the RBR family diversified very early in animals, to generate 10 subfamilies [11]. Moreover, not only the proteins of those subfamilies have very different primary sequences, but also they generally contain characteristic, subfamily-specific protein domains [5], [8], [10]. Another important difference is that many independent gene losses have been detected in particular animal lineages, such as insects, nematodes or urochordates, leading to a much reduced number of RBR genes in these species [11]. A functional hypothesis was put forward to explain why some genes were strictly conserved while others were often lost. Given that only a few RBR genes have broad patterns of expression and have strong effects on fitness, it is possible that, under the right circumstances (e. g. modifications of the functions of cell types or tissues, changes in the proteins to be ubiquitinated, etc), the rest may be lost without much trouble [11]. However, why precisely certain animal lineages and not others tend to reduce the number of RBR genes is still a mystery.

A clue of why plant and animal RBRs may be evolving differently is provided by the patterns of expression of *Arabidopsis* and *Oryza* RBR genes which have been described above. Although the data obtained are limited, the results are compatible with these species having two functionally different sets of RBR genes. One of them is formed by one or a few genes of each subfamily that have moderate to high levels of expression in many/all developmental times (Figures 6 and 7; top and middle panels). These genes may have essential, perhaps in some cases housekeeping, functions. The existence of this set of genes parallels perfectly what is found in animals, in which there is also a small set of broadly expressed genes in both humans and flies, which also belong to different subfamilies [11]. A second set, which is peculiar of plant species, consists in many genes that have a very low level of expression and sometimes, especially in *Oryza*, also a quite specific pattern of expression (Figures 6, 7; bottom panels). Significantly, many of those genes emerged by tandem duplications (see above). Nothing similar has been detected in animals. There are two possible explanations for this pattern. The first is somewhat trivial: the genes, which have appeared recently (given that they are not present in related species; Figures 4 and 5), may be in the process of becoming non-functional and disappearing. A second option is far more interesting and would fit better the fact that so many tandemly repeated genes have been produced in two distant relatives, *Arabidopsis* and *Oryza*, independently: the products of these genes may have particular functions in which microdifferentiation may be an advantage.

Several alternative reasons for that advantage may be hypothesized. A first possibility is suggested by results obtained by Rizzon et al. [30] and Hanada et al. [31]. They found a correlation between the presence of tandem duplicates in *Arabidopsis* and *Oryza* and the fact that the duplicates were involved in responses to stress or other environmental stimuli. It is unclear however to which kind of stimuli might those RBR genes be responding. For example, in the case of *Arabidopsis*, those stimuli should be species-specific (i. e. significant in *Arabidopsis* but, for example, not in *Brassica*, which lacks most of the duplicates) and perhaps specific for pollen (see data above). As indicated above, the available functional data, very scarce, does not suggest any easy explanation for this particular pattern. In any case, further exploration of the idea that RBR ubiquitin ligases were amplified to respond to some kind of stress, perhaps as part of a defensive system [32] may be rewarding. The evidence of overexpression of multiple housekeeping RBR genes after geminivirus infection [28], described above, is a first hint of what may be a promising line of research.

A second, alternative hypothesis would be that these plants require multiple related RBR proteins to cope with an increased diversity of substrates to be ubiquitinated in particular cells, tissues or developmental periods. Following this line of thought, there are three significant patterns that are emerging from the comparative analyses of families of proteins involved in ubiquitination in plants. First, several of them have followed patterns of rapid amplification, often by generation of tandem duplicates, similar to those described here [19]–[21], [33]–[35]: Second, these amplifications seem to go together with specialization of the expression patterns. This has been shown to occur in members of cullin ubiquitin ligase complexes, such as F-box proteins [24], [34] and Skp1-related proteins, in which moreover multiple genes seem to be expressed specifically in pollen [36]. Third, in general transcriptome analyses, an increased expression of multiple ubiquitination-related genes has been found in pollen, both in *Arabidopsis* (ref. [37]; sperm cells) and in soybean [38]. The parallelism of all these findings is very suggestive. Still, what kind of substrates may have generated these specific needs of many alternative, almost identical, ubiquitin ligases is impossible to predict with the available data. Further research will be necessary to establish this interesting point.

In summary, the study of plant RBR sequences have shed new light on the potential of diversification of this family of ubiquitin ligases and also opens interesting new views about how the ubiquitin system as a whole may be evolving differently in plants and animals. Future results in both plants and animals, as well as the analyses of other organisms, most especially fungi and protozoa, may offer additional insights about the evolution of this significant group of genes.

## Materials and Methods

I used as a starting point the database of 1174 aligned RBR protein sequences described in [11]. For this work, the 291 sequences in that database that belonged to viridiplantae species were selected. Additional analyses, following the same methods described in that work, were performed in March 2010, in order to add all the sequences made available by the sequencing projects since the previous study. 207 additional sequences were discovered. The final database therefore contained 498 RBR sequences.

Phylogenetic analyses were in general performed as in Marín [11]. Neighbor-joining (NJ) trees were characterized using MEGA 4 [39]. Maximum-parsimony (MP) trees were obtained using PAUP* 4.0, beta 10 version [40]. Finally, maximum-likelihood (ML) trees were obtained using PHYML 3.0 [41]. Details of the parameters used can be found in [11]. Minor changes to improve the analyses respect to that paper were as follows: 1) for MP, the maximum number of tied trees was increased from 20 to 100 and the tree-bisection-reconnection algorithm, which is more exhaustive and precise than the subtree pruning-regrafting method used in [11], was chosen. For ML, the improved Le and Gascuel matrix of amino acidic substitutions [42] was used instead of the older Blosum62 matrix. In all the analyses, 1000 bootstrap replicates were performed to establish the reliability of the NJ and MP trees. For ML, which is much more computer intensive, 200 bootstrap replicates were obtained. MEGA 4 was used to edit and draw the trees in Figures 1–5.

Structural searches were performed using the integrated tool InterProScan [43]. Searches for tandem repeated genes in *Arabidopsis thaliana* and *Oryza sativa japonica* were based on the information about the location of all genes of those species in their respective genomes, also available at the NCBI. Microarray data for *Arabidopsis thaliana* developmental samples (including some from characteristic mutants) were obtained using the AtGenExpress Visualization Tool (http://jsp.weigelworld.org/expviz/expviz.jsp; data obtained by [24]). Further *Arabidopsis* expression data were taken from the supplementary data accompanying the paper by Pina et al. [25] or directly from the Gene Expression Omnibus (GEO) webpage (http://www.ncbi.nlm.nih.gov/geo/) or the TAIR webpage (http://www.arabidopsis.org/). Expression data from *Oryza* were obtained from the Yale Virtual Center for Cellular Expression Profiling of Rice, which contains RiceAtlas (http://bioinformatics.med.yale.edu/riceatlas/; [26]). The unplanned comparisons among means of the levels of expression were performed using the GT2 method, as described in [44], which has the advantage of accomodating unequal sample sizes.
